# The association between inborn errors of immunity and Hodgkin lymphoma—A register‐based case–control study in Sweden

**DOI:** 10.1111/bjh.70743

**Published:** 2026-08-12

**Authors:** Agnes Winsa, Ingemar Lagerlöf, Fredrik Ellin, Mats Fredriksson, Åsa Nilsdotter‐Augustinsson, Marcus Bendtsen, Jörg Cammenga, Sofia Nyström

**Affiliations:** ^1^ Department of Clinical Immunology and Transfusion Medicine Region Östergötland Linköping Sweden; ^2^ Department of Biomedical and Clinical Sciences Linköping University Linköping Sweden; ^3^ Clinical Department of Haematology in Linköping Region Östergötland Linköping Sweden; ^4^ Department of Internal Medicine Kalmar County Hospital Kalmar Sweden; ^5^ Department of Clinical Sciences Lund University Lund Sweden; ^6^ Clinical Department of Infectious Diseases in Region Östergötland Norrköping Sweden; ^7^ Department of Health, Medicine and Caring Sciences Linköping University Linköping Sweden; ^8^ Department of Haematology Skåne University Hospital Lund Sweden; ^9^ Molecular Medicine and Division of Gene Therapy Lund University Lund Sweden

**Keywords:** epidemiology, Hodgkin lymphoma, inborn errors of immunity, risk factor

## Abstract

Immune dysfunction is implicated in the aetiology of Hodgkin lymphoma (HL), but the association between inborn errors of immunity (IEI) and HL remains poorly understood. We conducted a retrospective, register‐based case–control study in Sweden over five decades to investigate the relationship between IEI and HL. Individuals diagnosed with HL in the Swedish Cancer Register between 1969 and 2019 were matched with population controls. Data were linked to the National Patient Register to identify recorded the International Classification of Diseases (ICD)‐coded IEI diagnoses. Odds ratios (ORs) for HL associated with any IEI and IEI subgroups were estimated using conditional logistic regression. The study included 10 549 HL patients and 42 225 controls. Median age at HL diagnosis was 49 years, and 57% were male. Twenty‐two HL cases and 11 controls had a recorded IEI diagnosis; most were predominantly antibody deficiencies (PAD; 14 cases and 8 controls). Any IEI was associated with increased odds of HL (OR 8.0; 95% confidence interval [CI] 3.9–16.5). No clear differences in HL risk were observed between IEI subtypes. IEI was strongly associated with increased odds of HL. These findings support a role for IEI in HL pathogenesis and may suggest a distinct aetiological pathway for HL compared with other IEI‐associated lymphomas.

## INTRODUCTION

Hodgkin lymphoma (HL) is a mature B‐cell derived neoplasm characterized by a few malignant cells present within a microenvironment of inflammatory cells.^1^, ^2^ Inborn errors of immunity (IEI) are a heterogeneous group of genetic disorders with a prevalence of 6 per 10 000,^3^ characterized by increased susceptibility to infections, immune dysregulation, lymphoproliferation and cancer.^4^, ^5^ Patients with predominantly antibody deficiencies (PAD) appear particularly vulnerable to cancer with lymphoma being the most frequently observed malignancy.^6^, ^7^ PAD is marked by B‐cell dysfunction and impaired antibody production and shares a common B‐cell lineage origin with HL.^8^

Elevated risk of HL is associated with a history of rheumatoid arthritis, systemic lupus erythematosus, sarcoidosis and immune thrombocytopenia,^9^, ^10^, ^11^ all conditions referred to as immune dysregulation in IEI.^4^ Moreover, non‐malignant lymphoproliferation is common in IEI and shares clinical and immunophenotypic features with HL.^4^, ^12^ Impaired antibody production and infections in IEI are typically managed with immunoglobulin replacement and antibiotics,^4^ whereas immune dysregulation and lymphoproliferation often remain insufficiently treated.^4^, ^13^, ^14^ Patients with IEI are at increased risk of severe infections with Epstein–Barr virus (EBV),^15^ which has been proposed as a potential mediator of HL in autoimmune diseases.^10^, ^16^, ^17^ Additional mechanisms that may contribute to B‐cell carcinogenesis in IEI include lymphocyte maturation defects and deoxyribonucleic acid (DNA) repair deficiencies.^15^, ^18^, ^19^ An elevated concordance rate for HL among monozygotic twins suggests an underlying genetic susceptibility.^20^ Moreover, impaired T‐cell responses have been observed in healthy twins of individuals with HL, further implicating a link to IE.^21^ Several IEI‐associated germline variants also overlap with known HL predisposition genes, supporting the hypothesis of a shared genetic susceptibility.^22^ Immune dysfunction initially classified as acquired may, in some cases, reflect an underlying IEI, unmasked by severe treatment‐related infections or poor immune reconstitution post‐lymphoma treatment.^21^, ^23^ Investigating the risk of HL in IEI is motivated by the known risk factors associated with IEI and the possibility of shared genetic susceptibility. A better understanding of the association between IEI and HL may offer approaches to optimize treatment for patients with HL and early detection in patients with IEI.

In this nationwide study, the aim was to compare the odds for HL in patients diagnosed with IEI with a general population comparator cohort over five decades by linking National Swedish Registers. Furthermore, we aimed to examine IEI categories more strongly associated with HL.

## METHODS

### Study design and data sources

We performed a nationwide case–control study in Sweden using data from the following national registers with high coverage: the Total Population Register (TPR), the National Patient Register (NPR), the National Cancer Register (NCR) and the National Cause of Death Register (NCDR). The registers are further described in Supplementary Methods.

### Study population, exposure and outcome

The population of individuals diagnosed with HL was identified from the NCR during the years 1969–2019 using the International Classification of Diseases (ICD) 7 code 201. Using the TPR, four population‐based controls per HL case, matched by year of birth, sex and region of residence were randomly selected by Statistics Sweden. Swedish residents alive and without HL or any previous haematological malignancy, ICD 7 codes 203–207, on the index date were eligible for matching. For the entire cohort of HL cases and population‐based controls, the NPR was searched for ICD diagnosis codes indicative of IEI. Details on the classification, clinical heterogeneity and the evolving recognition of IEI, as well as changes in ICD coding systems for IEI over time, are provided in the Supplementary Methods. A full list of ICD 8–10 IEI codes is available in Table S1. Exposure was defined as the presence of an ICD diagnosis code for IEI in the NPR recorded more than 12 months prior to the study entry date, which was the date of lymphoma diagnosis for cases and the respective date for matched controls. To gain a broader understanding of the disease spectrum, we also extracted ICD codes potentially related to IEI including those associated with bacterial and viral infections, autoimmunity, inflammatory conditions and lymphoproliferation (Table S2). To minimize the risk of including cases of secondary immunodeficiency, individuals with an ICD code indicative of organ transplantation, human immunodeficiency virus (HIV) infection or acquired immunodeficiency syndrome (AIDS) were excluded from the study (Table S2). The outcome was defined dichotomously as either the occurrence or non‐occurrence of HL. Data were linked to the NCDR to identify individuals who were deceased. The cause of death was obtained by ICD codes categorized according to chapter, to examine patterns in cause‐specific mortality.

### Statistics

We estimated the association between IEI and HL using conditional logistic regression with risk sets defined by the matching variables (sex, age and county of residence). Details of models used and sensitivity analyses are available in Supplementary Methods.

### Ethical approval

The study was approved by the Swedish Ethical Review Authority (Dnr 2020‐05958), and given its register‐based nature, the requirement for individual informed consent was waived.

## RESULTS

### Study population and cohort characteristics

A total of 10 656 individuals with an ICD code of HL were identified in the NCR between 1969 and 2019. Of these, 107 individuals were excluded; 74 due to lacking a record in the TPR, which precluded the identification of matched controls, and 33 because they had an ICD code in the NPR indicating organ transplantation or HIV/AIDS (Figure 1). The population‐based controls consisted of 42 326 individuals. Of the control population, 101 cases were excluded because they had an ICD code in the NPR indicating organ transplantation or HIV/AIDS. Demographics of the study cohort are presented in Table 1. Median age at HL diagnosis was 49, interquartile range 28–69 years, with the typical biphasic distribution of age at diagnosis. Male sex predominated (57%) among individuals diagnosed with HL. The annual number of HL varied from 165 to 324, and the median calendar year of HL diagnosis was 1992 (Figure S1). Among the HL cases, a Systematized Nomenclature of Medicine‐Oncology, second edition (SNOMED‐O/2) morphology code in the Cancer Register was available for the 5163 individuals who were diagnosed with HL 1993 or later. The subtype distribution showed that 88% were classical Hodgkin lymphomas (cHL). The most common subtype was nodular sclerosis cHL (46%), followed by unspecified cHL (22%) and mixed cellularity cHL (16%). Lymphocyte‐rich and lymphocyte‐depleted cHL accounted for 3% and 1% of cases respectively. Nodular lymphocyte predominant HL constituted 4%, while 8% were classified as others (Figure S2).

**FIGURE 1:**
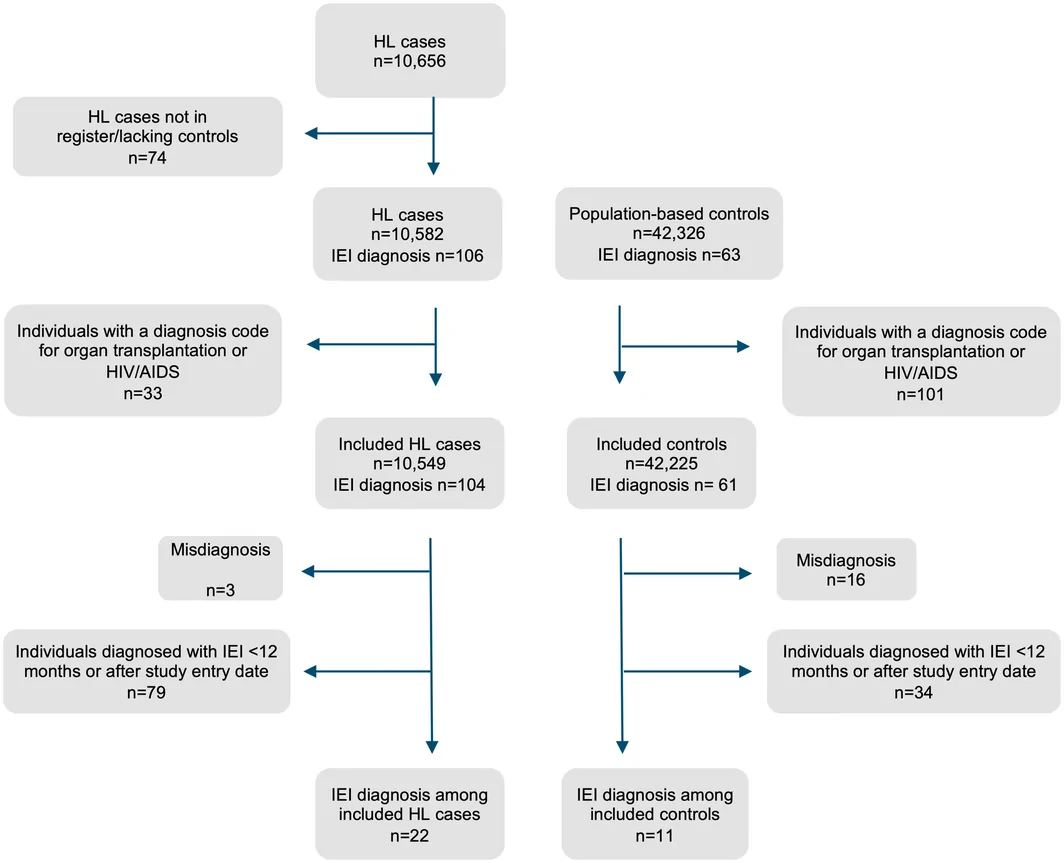
Flow chart of the study cohort. The study population included all individuals diagnosed with Hodgkin lymphoma in the National Cancer Register during the period 1969–2019. Population‐based controls, matched for sex, age and region of residency, were identified in the Total Population Register. Individuals with an ICD code indicative of organ transplant or HIV/AIDS in the National Patient Register as well as individuals not found in the Total Population Register were excluded from the study. Misdiagnosis refers to the ICD code D82.4A; allergic, IgE‐mediated reaction. HL, Hodgkin lymphoma; IEI, inborn errors of immunity.

**TABLE 1:** Characteristics of the total study cohort.

	HL population	Control population
Included individuals, *n*	10 549	42 225
Age, years median (IQR)	49 (28–69)	49 (28–69)
Diagnosis‐year, median (IQR)	1992 (1979–2007)	N/A

Variables	Categories	*n*	%	*n*	%
Cohort entry age	<15 years	332	3.1	1334	3.1
	15–24 years	1647	15.6	6603	15.6
	25–34 years	1680	15.9	6721	15.9
	35–44 years	1182	11.2	4731	11.2
	45–54 years	1010	9.6	4042	9.6
	55–64 years	1297	12.3	5195	12.3
	65–74 years	1702	16.1	6807	16.1
	>75 years	1699	16.1	6792	16.1
Cohort entry year	1969–1993	5564	52.7	22 257	52.7
	1994–2019	4985	47.3	19 968	47.3
Sex	Male	6004	56.9	24 036	56.9
	Female	4545	43.1	18 189	43.1

Abbreviations: HL, Hodgkin lymphoma; IQR, interquartile range; N/A, not applicable.

### Occurrence of IEI in the HL population

In total, 106 HL cases and 63 population‐based controls were diagnosed with an IEI. Twenty‐three HL cases and 16 controls had their first IEI diagnosis code recorded at least 12 months prior to the index date and were included in the analyses (Figure 1). Of the 39 individuals, one HL case and five controls were excluded due to likely IEI misdiagnosis (D82.4A; allergic, immunoglobulin (Ig) E‐mediated reaction rather than Hyper IgE syndrome) rendering a total of 22 HL cases and 11 controls. The frequency of individuals with an IEI diagnosis more than 12 months prior to study entry date was approximately 200 per 100 000 individuals among HL cases and 26 per 100 000 among controls. Median age at IEI diagnosis was 45 years (range 0–79) and male sex was more common (60%) among individuals diagnosed with IEI and HL (Table 2). The median age at HL diagnosis of individuals diagnosed with IEI was 47 (range 6–83) years. Predominantly antibody deficiency (PAD) was the most common IEI category in the HL, as well as in the control population (Table 2). A detailed list of IEI ICD diagnosis codes in the study population according to the International Union of Immunological Societies (IUIS) category^8^ is available in Table S3. The median calendar year of HL diagnosis among individuals with a prior IEI was 2012 (Table 2). Eighteen of 22 individuals with IEI were diagnosed with HL in 1994 or later, and all individuals with IEI in the control population entered the study 1994 or later (Table 2). During the later half of the study period, 1994–2019, the frequency of individuals with an IEI diagnosis more than 12 months prior to study entry was approximately 280 per 100 000 individuals among HL cases and 55 per 100 000 among controls (Table 2).

**TABLE 2:** Characteristics of individuals diagnosed with inborn errors of immunity.

	HL population, *n* = 22	Control population, *n* = 11
Calendar year of IEI diagnosis, median (range)	2006 (1977–2013)	2006 (1994–2016)
Years of age at study entry, median (range)	47 (6–83)	40 (17–86)

Variables	Category	*n* (%)	*n* (%)
Cohort entry age	<15 years	3 (14)	0
	15–24 years	3 (14)	4 (36)
	25–34 years	2 (9)	0
	35–44 years	2 (9)	2 (18)
	45–54 years	3 (14)	0
	55–64 years	2 (9)	1 (9)
	65–74 years	5 (23)	1 (9)
	≥75 years	2 (9)	3 (27)
Cohort entry year, *n* (%)	1969–1993	4 (18)	0
	1994–2019	18 (82)	11 (100)
Frequency of IEI per 100 000	1969–2019	200	26
	1994–2019	280	55
Sex, *n* (%)	Male	13 (59)	6 (55)
	Female	9 (41)	5 (45)
IEI category, *n* (%)	PAD	14 (64)	7 (64)
	Other	8 (36)	4 (36)

Abbreviations: HL, Hodgkin lymphoma; IEI, inborn errors of immunity; PAD, predominantly antibody deficiency.

### Association between IEI and Hodgkin lymphoma

The odds of HL were estimated to be higher among individuals with IEI diagnosis compared with those without (OR 8.0; 95% confidence interval [CI] 3.9–16.5), Model I (Figure 2). When examining IEI subtypes, it was not possible to identify a statistically significant difference in the odds for HL between individuals without PAD versus individuals with PAD (OR 0.7; 95% CI 0.1–3.2), Model II (Figure 2). Including only cases and controls with index date 1994 or later yielded similar results (OR 6.5; 95% CI 3.1–13.9) and (OR 0.5; 95% CI 0.09–2.37) Model I and Model II respectively. Using all available IEI diagnosis dates also generated estimates comparable to those from the primary analyses. In sensitivity analyses using longer lag periods (≥2‐year and ≥5‐year) between IEI diagnosis and HL diagnosis, the association between IEI and HL remained robust (Table S4).

**FIGURE 2:**
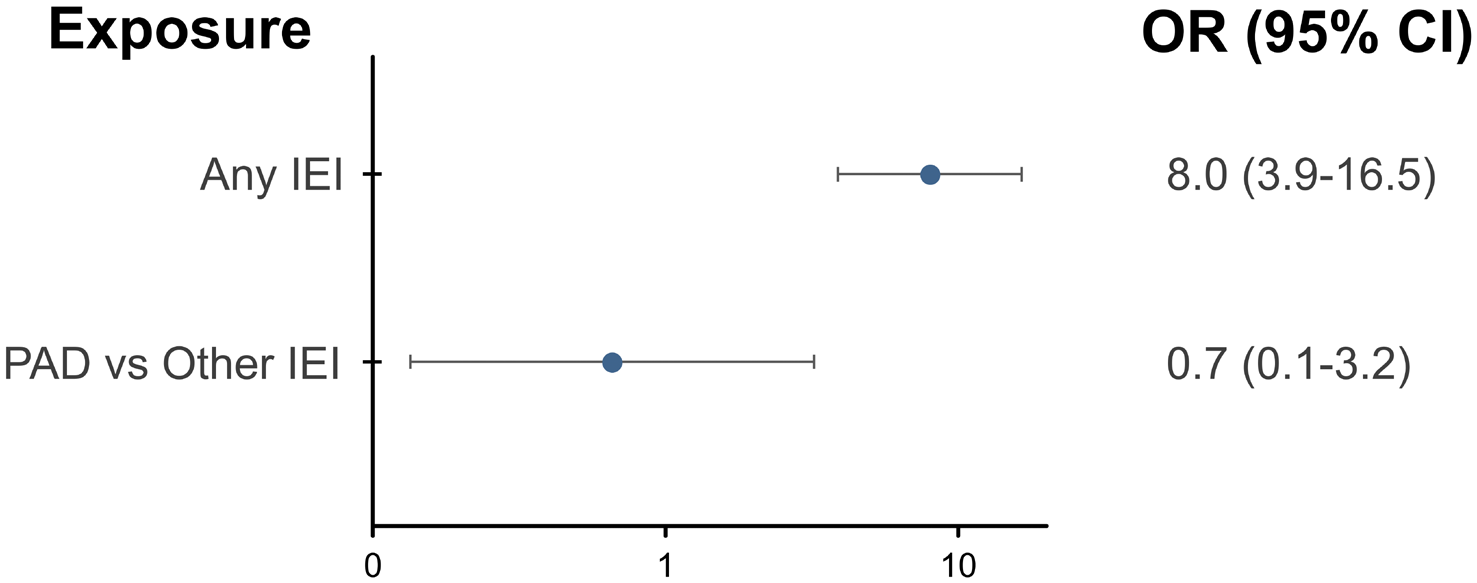
Inborn errors of immunity as a risk factor for Hodgkin lymphoma. Odds ratios for HL associated with IEI. HL, Hodgkin lymphoma; IEI, inborn errors of immunity; PAD, predominantly antibody deficiencies.

### Characteristics of patients with IEI and Hodgkin lymphoma

The distribution of age at HL diagnosis among individuals with IEI was comparable to that of the overall HL population. However, IEI diagnoses were concentrated in more recent calendar years (Figure S1A,B). Twenty‐four of 33 individuals with IEI had their first IEI ICD code appearing in the NPR 2000 or later (Figure 3A). Within the control population, one individual was diagnosed during 1994 and within the HL population five individuals were diagnosed during the 1990s and one during the 1980s and 1970s respectively. For individuals within the HL population diagnosed with IEI, the median age at IEI diagnosis was 41, range 0–79 years (Table 3). The time between IEI and HL ranged between 13 and 312 months (Table 3, Figure 3B). At the end of the study period, 12 (55%) of individuals with IEI in the HL population, and seven (64%) in the control population were still alive (Table 3, Figure 3B). The underlying condition causing death in the HL population was neoplasm (*n* = 8) or immunity disorder (*n* = 2), according to the NCDR (data not shown). Look‐back analysis in the NPR for IEI‐related diagnoses (Table S2) prior to HL diagnosis or cohort entry date showed that 32% of individuals with IEI were affected by bacterial infections prior to HL diagnosis (Table 3). Three individuals had a record of EBV or cytomegalovirus (CMV) infections prior to HL diagnosis, and four individuals had a history of autoimmunity. A record of inflammatory disease was found for five individuals, and one individual had been diagnosed with splenomegaly more than 10 years prior to HL diagnosis (Table 3). Inflammatory disease was found in 5 of the 11 controls diagnosed with IEI, but there was no record indicating a history of EBV or CMV infection. Among individuals with IEI in the HL population five had a record of two or more IEI‐related diagnose categories more than 1 year prior to HL diagnosis; within the control population the corresponding number was also five (Table 3). Eighteen of the 22 HL cases with a preceding IEI were diagnosed from 1993 onward, and for these cases, SNOMEDO/2 morphology codes were available. The subtype distribution was comparable to that in the overall HL population, with cHL nodular sclerosis type or not specified constituting the majority of cases (Table 3).

**FIGURE 3:**
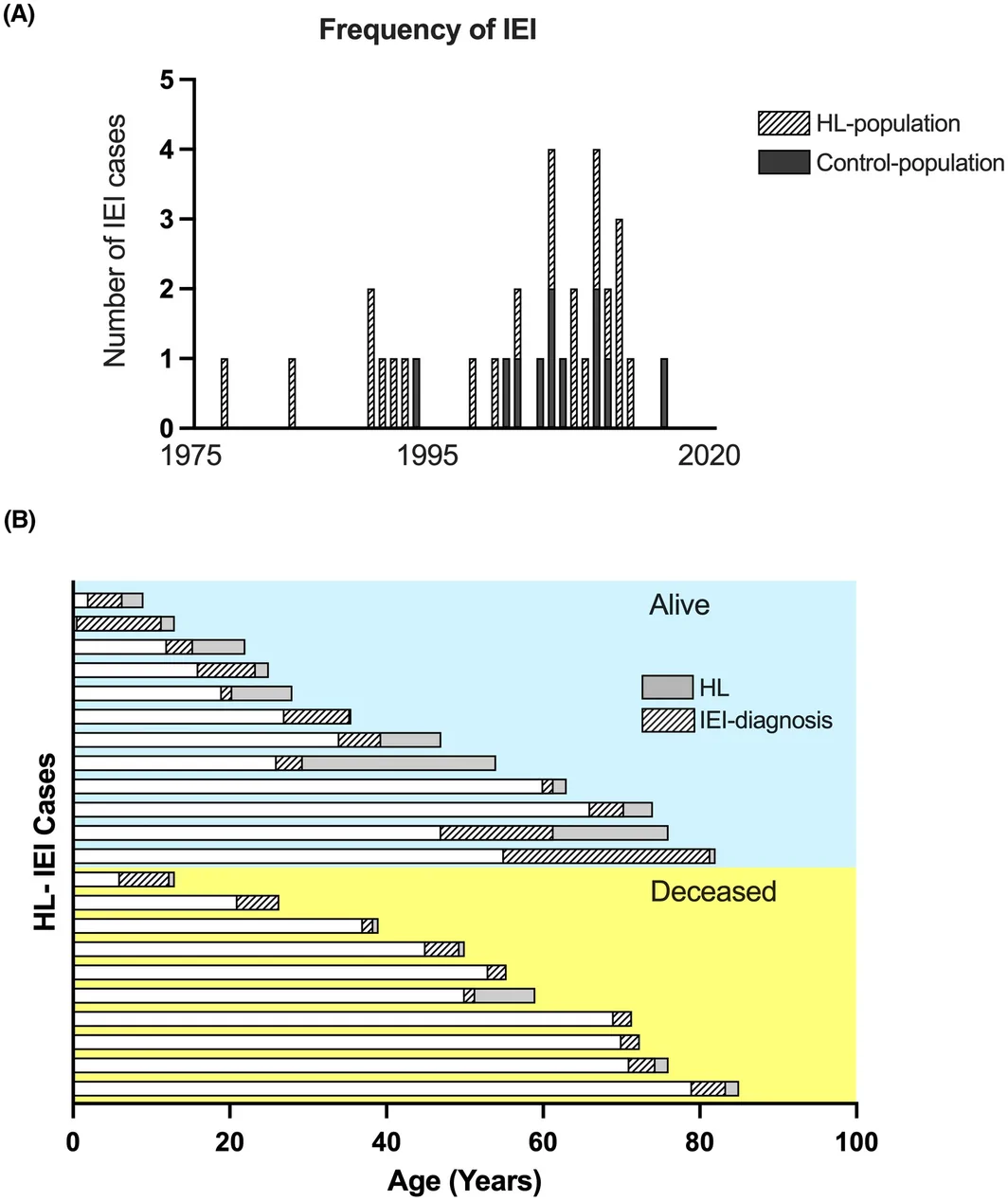
Time interval between diagnosis of inborn error of immunity and Hodgkin lymphoma. Year of first IEI ICD code in the National Patient Register of individuals diagnosed with HL and in population controls (A). Swimmer plot showing the age at diagnosis of IEI and the age at diagnosis of HL in individuals diagnosed with IEI prior to HL (B). Individuals alive at study end highlighted by blue colour, and deceased individuals by yellow colour. HL, Hodgkin lymphoma; IEI, inborn errors of immunity.

**TABLE 3:** Characteristics of individuals diagnosed with inborn errors of immunity and Hodgkin lymphoma.

	HL population, *n* = 22	Control population, *n* = 11
Age at IEI diagnosis, years, median (range)	41 (0–79)	33 (6–75)
Months between IEI and HL diagnosis, median (range)	50 (13–312)	N/A
Alive at study end, *n* (%)	12 (55)	6 (55)
IEI‐related diagnoses prior to HL diagnosis or cohort entry		
Bacterial infection,^a^ *n* (%)	7 (32)	4 (36)
Viral infection,^b^ *n* (%)	3 (14)	‐
Autoimmunity,^c^ *n* (%)	4 (18)	3 (27)
Inflammatory disease,^d^ *n* (%)	5 (23)	5 (45)
Lymphoproliferation,^e^ *n* (%)	1 (4.5)	1 (9)
0–1 IEI‐related diagnostic categories, *n* (%)	17 (73)	6 (55)
≥2 IEI‐related diagnostic categories, *n* (%)	5 (23)	5 (45)
HL subtypes		
Individuals with a SNOMED‐O/2 code, *n*	18	N/A
Classical HL, NOS, *n* (%)	6 (33%)	N/A
Classical HL, nodular sclerosis, *n* (%)	7 (39%)	N/A
Classical HL, mixed cellularity, *n* (%)	3 (17%)	N/A
Classical HL, lymphocyte rich, *n* (%)	1 (6%)	N/A
Classical HL, lymphocyte depleted, *n* (%)	0 (0%)	N/A
Nodular lymphocyte predominant HL, *n* (%)	1 (6%)	N/A

Abbreviations: HL, Hodgkin lymphoma; IEI, inborn errors of immunity; N/A, not applicable; NOS, not otherwise specified; SNOMED‐O/2, Systematized Nomenclature of Medicine‐Oncology, second edition.

^a^ Pneumonia, sinusitis, bronchiectasis and bacterial gastroenteritis.

^b^ Mononucleosis, and CMV infection.

^c^ Coeliac disease, immune‐mediated thrombocytopenia/Evans syndrome.

^d^ Rheumatoid arthritis, HLH, inflammatory bowel disease and psoriasis.

^e^ Splenomegaly and lymphadenopathy.

## DISCUSSION

In this nationwide register‐based case–control study spanning five decades, we demonstrate that a prior diagnosis of IEI was associated with substantially increased odds of HL The occurrence of any IEI among individuals with HL was approximately eightfold higher than in matched population controls. Furthermore, the distribution of histological subtypes of HL among individuals with a preceding IEI diagnosis mirrored that of the overall HL population, with nodular sclerosis being the most frequent subtype. The results support a strong association of IEI and cHL.

Our findings regarding the characteristics of the study population were in line with current knowledge regarding the epidemiology of HL.^2^ The distribution of patients reflected the expected frequency of HL in western countries, with a predominance of male patients and a bimodal distribution of age at diagnosis. Furthermore, the morphological subtypes, classified according to SNOMEDO/2, were consistent with previously reported patterns. The concordance between our cohort characteristics and previously reported epidemiological patterns supports the representativeness of the study population. As expected, we saw an increased frequency of IEI among HL cases compared to controls, when looking at occurrence at any time during the study period. Importantly, the difference remained when we excluded dates within 12 months prior to HL diagnosis or IEI diagnosis after HL diagnosis, where treatment otherwise could have been causative. Additional analyses using longer lag periods (≥2 and ≥5 years) showed consistent associations, supporting that the observed association is not driven by temporally proximate diagnoses. The findings support the hypothesis that IEI may play a role in the pathogenesis of HL.

To the best of our knowledge, this is the first study to use population‐based data to investigate the association between IEI and HL. The increased risk of lymphoma among patients with PAD has long been recognized.^14^, ^24^, ^25^ However, reports detailing World Health Organization classifications of lymphoma subtypes in this patient group are largely lacking. Studies based on cohorts of patients with IEI in general, PAD or more specifically common variable immunodeficiency (CVID), or PAD with a CVID‐like phenotype,^5^ have reported that 5%–32% of lymphoma cases were classified as classic HL.^25^, ^26^, ^27^, ^28^, ^29^ In one of these studies, HL subtypes were reported and were predominantly of the mixed cellularity type.^29^ This contrasts with our findings, in which nodular sclerosis was the most common subtype. Despite the strong relative association observed, the absolute risk is still likely to be low among individuals with IEI, given the rarity of HL in the general population.

It is likely that the underlying biological mechanisms are shared between lymphomas arising in IEI and those developing in other settings of immune deficiency and immune dysregulation. Proposed mechanisms contributing to lymphoma risk include chronic inflammation, viral infections, impaired tumour immune surveillance and genetic factors.^18^, ^30^ In patients with PAD, lymphoma is frequently reported to be preceded by non‐malignant lymphoproliferative manifestations. However, these observations may not be directly generalizable to HL, representing a minority of all lymphoma cases in this population.^31^, ^32^, ^33^ In the present study, only one of 14 patients with PAD had a history of preceding lymphoproliferation, suggesting that mechanisms may differ between lymphoma subtypes. Under‐ascertainment may also contribute to the low prevalence of lymphoproliferation in the present cohort, as lymphoproliferation is not consistently ICD‐coded, may be attributed to the underlying IEI, or misclassified as other conditions. Furthermore, IEI‐associated HL occurred at a similar age as in the total HL population, in contrast to previous studies reporting an earlier age at diagnosis for IEI‐associated lymphoma compared with lymphoma in the general population.^29^ Temporal restrictions of the study may have influenced the observed age distribution. However, the IEI‐associated HL cohort included a higher proportion of younger patients, suggesting that the similarity in age at diagnosis may partly reflect the limited number of cases.

EBV infection is likely to confer a risk for HL since the virus is harboured in the tumour cells of some patients.^34^ Primary immune‐mediated thrombocytopenia (ITP) is associated with an increased risk of HL.^9^, ^11^, ^35^ In addition, an elevated risk of HL has been reported among individuals with a prior history of bacterial sinusitis.^17^ Many individuals with IEI who developed HL were affected by at least one of the aforementioned conditions. Nevertheless, the overlap between genes frequently dysregulated in HL and genes implicated in IEI suggests that the underlying IEI may represent the primary pathogenic process.^2^, ^5^ This interpretation is supported by the high frequency of germline and somatic mutations identified in patients with IEI and lymphoma.^36^

In the present study, PAD accounted for the largest proportion of HL cases, most likely reflecting their higher overall prevalence.^4^ Nevertheless, the intrinsic risk of HL may be even higher in other, rarer disorders, such as DNA repair defects,^25^, ^37^ as well as in IEI associated with a high risk of developing EBV‐associated lymphoproliferative disorders.^38^ Activated phosphoinositide 3‐kinase delta syndrome (APDS) is a recently described PAD, first recognized in 2013.^39^ APDS is characterized by a markedly increased intrinsic risk of B‐cell malignancies possible due to dysregulation of the PI3Kδ pathway.^25^, ^40^ Infections with EBV and other herpesviruses are common in APDS and may further increase the risk of lymphoma.^28^ Our cohort is historical and evaluation for genetic causes of IEI was not performed during much of the study period. As a result, monogenic IEI remains unrecognized.

Interestingly, there was a marked increase in IEI diagnoses over time, with the majority occurring after 1994. We believe this mirrors increasing knowledge and diagnostic possibilities, rather than an actual increase in incidence of IEI diagnoses.^41^ The relatively high age at IEI diagnosis, particularly in the HL cohort, may similarly reflect delayed recognition of IEI earlier in the study period. More frequently recognized IEI in individuals who later developed HL may have contributed to the large difference in earliest year of IEI diagnoses between the HL and the control population, before 1994 when IEI ascertainment was limited. Establishing a definitive diagnosis of HL in patients with IEI can be challenging due to sparsely distributed malignant cells in biopsies and the presence of comorbidities such as non‐malignant lymphoproliferation, inflammatory conditions and viral infections, all of which may mimic emerging lymphoma.^42^ At present, lymphoma treatment in patients with IEI generally follows protocols designed for immunocompetent individuals, despite an increased risk of treatment‐related toxicity.^18^ Our findings demonstrate that patients with IEI are at increased risk of developing HL and underscore the importance of heightened clinical awareness and tailored therapeutic approaches, which may facilitate more personalized management and ultimately improve outcomes in this vulnerable patient population.

The Swedish national health registers are of high quality and offer nearly complete population coverage, enabling long‐term follow‐up.^24^, ^43^, ^44^, ^45^ Random selection of the matched comparison cohort, together with inclusion of all patients diagnosed with HL, reduces the risk of selection bias. The population‐based design enhances the representativeness of the findings and supports their generalizability to countries with a similar ethnic composition. The present study has limitations, primarily related to potential information bias and unmeasured residual confounding such as socioeconomic factors, number of siblings and a complete medical history. In addition, the low number of IEI cases among patients with HL, as well as among population controls, precluded adjusted analyses and direct comparisons of IEI cases between these two groups. Moreover, the long study period includes changes in HL classification, and some misclassification in earlier decades cannot be excluded. However, validation studies suggest reasonable diagnostic accuracy even in these periods.^46^

In conclusion, our findings demonstrate a statistically significant association between IEI and HL, suggesting a potential contribution of IEI to lymphomagenesis. Moreover, our results point to an aetiology of HL that differs from other lymphoma subtypes arising in the context of IEI.

## AUTHOR CONTRIBUTIONS

**Mats Fredriksson:** Conceptualization; methodology; writing – review and editing. **Åsa Nilsdotter‐Augustinsson:** Investigation; conceptualization; writing – review and editing. **Agnes Winsa:** Conceptualization; data curation; formal analysis; writing – original draft; project administration; investigation; visualization. **Sofia Nyström:** Conceptualization; methodology; supervision; funding acquisition; investigation; resources; writing – review and editing; visualization. **Fredrik Ellin:** Conceptualization; writing – review and editing. **Ingemar Lagerlöf:** Conceptualization; supervision; writing – review and editing; investigation. **Jörg Cammenga:** Conceptualization; methodology; funding acquisition; writing – review and editing. **Marcus Bendtsen:** Methodology; formal analysis; supervision; writing – review and editing; validation.

## FUNDING INFORMATION

This study was supported by research funding from FORSS, Medical Research Council of Southeast Sweden (FORSS‐1033520) and ALF Grants, Region Östergötland, Sweden (RÖ‐1033512) to Sofia Nyström.

## CONFLICT OF INTEREST STATEMENT

Sofia Nyström received consultancy fees from Takeda and Grifols outside of this work. All other authors declare no conflicts of interest.

## CLINICAL TRIAL REGISTRATION

Researchweb.org, ro‐1037887.

## Supporting information


**Figure S1.** Distribution of Hodgkin lymphoma cases by birth year and calendar year of diagnosis. Birth year distribution of all HL cases (grey bars). Arrows indicate individuals diagnosed with IEI prior to HL (A). Calendar year of HL diagnosis. Grey bars represent all HL cases, and black bars indicate cases with prior IEI (B). This figure is intended to illustrate the temporal distribution of cases and the small number of IEI associated HL cases across both birth cohorts and diagnostic periods. HL, Hodgkin lymphoma; IEI, inborn errors of immunity.

**Figure S2.** Pie chart of Hodgkin lymphoma subtypes. Within the Hodgkin lymphoma (HL) population a SNOMED‐O/2 category was available for 49% (*n* = 5163) of the individuals, that is individuals with study entry 1993 or later. cHL, classical Hodgkin lymphoma; NOS, not otherwise specified.

**Table S1.** Full list of ICD 8–10 inborn errors of immunity codes.
**Table S2.** Full list of ICD 8–10 codes of diagnoses related to inborn errors of immunity.
**Table S3.** Number of individuals per IUIS IEI category in the total cohort.
**Table S4.** Odds ratio for HL associated with prior IEI diagnosis, stratified by time interval between IEI diagnosis and HL diagnosis.

**Data S1.** Supplementary methods.

## Data Availability

The data that support the findings of this study are available on request from the corresponding author. The data are not publicly available due to privacy or ethical restrictions.
