# Supplementary material for: The association between inborn errors of immunity and Hodgkin lymphoma—A register‐based case–control study in Sweden
Source: Br J Haematol. 2026 Aug 12;209(4):1629–37. doi: 10.1111/bjh.70743 (PMC13674171; doi:10.1111/bjh.70743)
Supplement: Supplementary file 1 [file BJH-209-1629-s002.docx]

**Supplementary Tables S1-S4**

**Table S1. Full list of ICD 8-10 inborn errors of immunity codes**

| **ICD version (years)** | **ICD code** | **Diagnosis** |
| --- | --- | --- |
| ICD 8  (1969-1986) | 275.00 | Agammaglobulinemia |
|  | 275.10 | Hypogammaglobulinemia |
| ICD 9  (1987-1996) | 288.1 | Chronic granulomatous disease |
|  | 279.0 | Deficiency of humoral immunity |
|  | 279.1 | Deficiency of cell-mediated immunity |
|  | 279.2 | Combined immunodeficiency |
|  | 279.3 | Unspecified immunodeficiency |
|  | 279.8 | Other specified disorders of immune mechanism |
|  | 279.9 | Unspecified disorder of immune mechanism |
| ICD 10  (1997- ) | D71 | Chronic granulomatous disease |
|  | D80 | Immunodeficiency with predominantly antibody defects |
|  | D81 | Combined immunodeficiency |
|  | D82 | Immunodeficiency associated with other major defects |
|  | D83 | Common variable immunodeficiency |
|  | D84 | Other immunodeficiency disorders |

**Table S2. Full list of ICD 8-10 codes of diagnoses related to inborn errors of immunity**

| **ICD 8** | **ICD 9** | **ICD 10** |
| --- | --- | --- |
| *Septicemia* | | |
| 038.00 | 038.0 | A40 |
| 038.10 | 038.1 | A41 |
| 038.20 | 038.2 |  |
| 038.88 | 038.3 |  |
| 038.99 | 038.4 |  |
|  | 038.8 |  |
|  | 038.9 |  |
| *CNS infections* | | |
| 320 | 320 | G00 |
| 323 | 321 | G01 |
| 322 | 322 | G02 |
|  | 323 | G03 |
|  | 324 | G04 |
|  |  | G05 |
|  |  | G06 |
| *CNS inflammation* | | |
| 340.9 | 340 | G35 |
| 341 | 341 | G61 |
| 354 | 357.0 | H20 |
| 364 | 364.2 | H46 |
|  | 377.3 |  |
| *Respiratory infections* | | |
| 480.9 | 480 | J12 |
| 484.9 | 481 | J13 |
| 481.9 | 482 | J14 |
| 482 | 483 | J15 |
| 483.9 | 484 | J17 |
| 486 | 486 | J18 |
| 513.9 | 513 | J85 |
| *Respiratory inflammation* | | |
| 503 | 473 | J32 |
| 491 | 491.9 | J42 |
| 493.00 | 491.2 | J42 |
| 518.99 | 493 | J45 |
| 519.20 | 494 | J47 |
| 519.21 | 515 | J84 |
| 519.22 | 516 | J96 |
| 519.25 | 491.0 |  |
| 519.28 | 517.0 |  |
| 517.01 | 446.2 |  |
| *Gastrointestinal infections* | | |
| 001 | 002 | A02 |
| 002 | 003 | A07.0 |
| 003 | 007.0 | A07.1 |
| 007.00 | 007.1 | A07.2 |
| 007.10 | 007.2 | A07.3 |
| 007.96 | 007.3 | A07.8 |
| 007.97 | 007.8 | A07.9 |
| 007.99 | 007.9 | K63.0 |
| 007.20 | 569.0 |  |
| 006 |  |  |
| 569.00 |  |  |
| *Gastrointestinal inflammation* | | |
| 563.00 | 555 | K50 |
| 563.10 | 556 | K51 |
| 569.02 | 558 | K52.8 |
| 561 |  | K52.9 |
| 564.10 |  |  |
| *Gastrointestinal disorders* | | |
| 536.01 | 535.0 | K29.4 |
| 536.02 | 564.0 | K90.4 |
| 269.00 | 564.9 | K90.0 |
| 269.10 | 579.0 | K90.9 |
| 564.19 | 579.1 |  |
|  | 579.9 |  |
|  | 564.1 |  |
| *Liver diseases* | | |
| 571.90 | 571.2 | K72.9 |
| 571.98 | 573.3 | K76.6 |
| 571.99 | 571.5 | K75.3 |
| 572.99 | 572A | K75.0 |
| 573 | 571.8 | K75.4 |
|  | 571.9 | K75.8 |
|  | 572.2 | K75.9 |
| *Biliary tract diseases* | | |
| 575.01 | 571.6 | K83.0 |
| 575.03 | 576.0 | K83.8 |
| 575.04 | 576.1 | K83.9 |
| 575.05 | 576.8 | K74.3 |
|  | 576.9 |  |
| *Kidney/urinary tract diseases* | | |
| 580.99 | 446.0 | N00 |
| 593.10 | 580 | N01 |
| 581.99 | 583.8 | N04 |
|  | 580 | N05 |
|  | 581 |  |
| *Skin disorders* | | |
| 696 | 696.1 | L40 |
| 704.00 | 696.8 | L40.5 |
| 704.01 | 703.8 | L60.5 |
| 709.05 | 704.0 | L63 |
| 695.40 | 709.0 | L80.9 |
|  | 695.4 | L93 |
| *Systemic inflammatory diseases* | | |
| 712 | 696.0 | J99 |
| 446.20 | 714 | M05 |
| 446.98 | 446.1 | M06 |
| 446.00 | 446.0 | M07 |
| 446.10 | 710.3 | M08 |
| 716.00 | 710.4 | M30.1 |
| 716.10 | 710.1 | M30.3 |
| 734.00 | 710.0 | M31 |
| 734.01 | 710.8 | M32 |
| 734.09 | 710.9 | M33 |
| 734.10 | 720 | M34 |
| 734.91 | 446.3 | M35 |
| 734.98 | 135 | M45 |
| 734.99 |  | D86 |
| 135 |  |  |
| 287.00 |  |  |
| *Autoimmune diseases* | | |
| 245 | 245.2 | E06.3 |
| 250 | 250 | E10 |
|  | 279.3 |  |
| *Herpes virus* | | |
| 079.51 | 078.5 | B25 |
| 075 | 075 | B27 |
| *Hematological disorders* | | |
| 283.90 | 283.0 | D59.1 |
| 283.91 | 287.0 | D69.3 |
| 287.10 | 287.1 | D70 |
| 287.11 | 287.2 | D72.8 |
| 287.19 | 288.0 |  |
| 288.99 | 288.9 |  |
| *Enlargement of organs or lymph nodes* | | |
| 785.10 | 289.5 | D73.1 |
| 782.80 | 789.5 | D76 |
| 289.10 | 789.6 | R16 |
| 289.20 | 289.1 | R59 |
| 289.30 | 289.2 |  |
| 782.70 | 289.3 |  |
|  | 785.5 |  |
|  | 446.0 |  |

**Table S3. Number of individuals per IUIS IEI category in the total cohort**

| **Version** | **Code** | **Diagnosis** | **HL (n=22)** | **Non-HL (n=11)** |
| --- | --- | --- | --- | --- |
| *Predominantly antibody deficiency* | | | | |
| ICD8  (1969-1986) | 275.0 | Agammaglobulinemia | 0 | 0 |
|  | 275.1 | Hypogammaglobulinemia | 2 | 0 |
| ICD9  (1987-1996) | 279.0 | Deficiency of humoral immunity | 5 | 1 |
| ICD10  (1997- ) | D80 | Immunodeficiency with predominantly antibody defects* | 6 | 5 |
|  | D83 | Common variable immunodeficiency | 1 | 1 |
| *Combined immunodeficiencies with associated or syndromic features* | | | | |
| ICD8 |  | N/A |  |  |
| ICD9 |  | N/A |  |  |
| ICD10 | D82 | Immunodeficiency associated with other major defects**^§^** | 3 | 3 |
| *Other* | | | | |
| ICD8 |  | N/A |  |  |
| ICD9 | 279.3 | Unspecified immunodeficiency | 0 | 0 |
|  | 279.9 | Unspecified disorder of immune mechanism | 0 | 0 |
| ICD10 | D84 | Other immunodeficiency disorders**^#^** | 5 | 1 |

IUIS, International Union of Immunological Societies; IEI, inborn errors of immunity; ICD, International classification of disease; HL, Hodgkin lymphoma population; non-HL, population-based controls; N/A, not applicable; *HL, IgA/IgG-subclass deficiency (n=1), hereditary hypogammaglobulinemia (n=2), IgG-subclass deficiency (n=2), X-linked agammaglobulinemia (n=1), non-HL, IgA deficiency (n=2), IgA/IgG-subclass deficiency (n=1), IgG-subclass deficiency (n=1), hereditary

hypogammaglobulinemia (n=1); **^§^**HL, DiGeorge syndrome (n=1), Wiskott-Aldrich’s syndrome (n=1), hyper IgE syndrome (n=1), non HL, Immunodeficiency with short-limbed stature (n=1), DiGeorge syndrome (n=1), other (n=1); **^#^**HL, bacterial infection (n=1), history of EBV/CMV infection (n=2), celiac disease (n=1), agranulocytosis (n=1), non-HL, C1 esterase inhibitor deficiency (n=1)**.**

**Table S4. Odds ratio for HL associated with prior IEI diagnosis, stratified by time interval between IEI diagnosis and HL diagnosis**

|  | Diagnosis of IEI, time prior to HL diagnosis (or study entry date) | | |
| --- | --- | --- | --- |
|  | ≥1 year | ≥2 years | ≥5 years |
| OR  (95% CI) | 8.0  (3.9-16.5) | 6.4  (2.9-14.1) | 6.0  (2.1-16.9) |

HL, Hodgkin lymphoma; IEI, inborn errors of immunity; OR, odds ratio; CI, confidence interval
