# Supplementary material for: The association between inborn errors of immunity and Hodgkin lymphoma—A register‐based case–control study in Sweden
Source: Br J Haematol. 2026 Aug 12;209(4):1629–37. doi: 10.1111/bjh.70743 (PMC13674171; doi:10.1111/bjh.70743)
Supplement: Supplementary file 2 [file BJH-209-1629-s004.docx]

**Supplementary Methods**

*Data sources*

The Total Population Register (TPR) is maintained by Statistics Sweden and contains data on date of birth, death, sex, and region of residency for the Swedish population. The National Patient Register (NPR) was established in 1964, achieved nationwide coverage in 1987, and contains data on all hospital inpatient admissions as well as on outpatient visits in specialist care from 2001 onward. The register has been found to be of high standard, with 99% coverage of somatic diagnoses [1]. The National Cancer Register (NCR) started in 1958 and covers the entire population, and it is mandatory for health-care providers to report new malignant disease to the register. From 1993 onward, morphologic coding according to the Systematized Nomenclature of Medicine - Oncology, second edition (SNOMED-O/2) was implicated in the NCR [2]. The National Cause of Death Register (NCDR) is an almost complete register of all deaths in Sweden since 1952, maintained by the Swedish National Board of Health and Welfare [3]. The study population was identified from the NCR and data were linked to the remaining registers by the Swedish National Board of Health and Welfare using the unique personal identification numbers assigned to all individuals in Sweden at birth or immigration [4].

*Identification and classification of individuals with inborn errors of immunity*

Inborn errors of immunity (IEI) comprise a rapidly expanding and heterogeneous group of disorders with diverse genetic causes, variable penetrance, and overlapping clinical phenotypes [5]. Advances in next-generation sequencing have markedly increased the number of recognised IEI [6]. Reflecting this, the European Society for Immunodeficiencies (ESID) registry has expanded from 2,386 patients in 2007 to 30,628 today, illustrating increased recognition over the past two decades [7,8]. IEI are currently classified into ten categories based on clinical, genetic, and immunological features, of which predominantly antibody deficiencies (PAD) constitute the largest group [6]. The genetic basis remains unknown in most PAD, and classification is largely based on clinical criteria. Common variable immunodeficiency (CVID), the most clearly defined PAD, remains a heterogeneous disorder characterised by hypogammaglobulinemia and frequently associated with immune dysregulation, including autoimmunity, lymphocytic infiltration, lymphoid hyperplasia, and lymphoma [8]. Over the period of the present study (1969–2019), the recognition of IEI in the International Classification of Diseases (ICD) has evolved substantially. Early ICD versions (1965–1986) included only limited categories, whereas ICD-9 introduced broader groups reflecting improved understanding of immune defects. ICD-10 provides more granular classification, including a specific code for CVID (Table S1). For this study, IEI were grouped into three categories based on IUIS classification criteria [6], with corresponding ICD codes assigned accordingly (Table S3).

*Statistics*

In our first model (Model I), inborn error of immunity (IEI) was modelled as a binary exposure (no IEI = 0 vs any IEI = 1) to estimate its overall association with HL. To estimate differences between IEI subtypes, we next extended Model I with a binary covariate representing predominantly antibody deficiencies (no PAD = 0 vs PAD = 1). In the latter model, Model II, the coefficient for the IEI covariate represents the odds ratio of HL associated with IEI and no PAD, while the coefficient for the PAD covariate represents the relative difference in the odds ratio of HL associated with IEI and PAD versus IEI and no PAD. As sensitivity analyses, we estimated Model I and Model II using (i) only cases and controls with an index date in 1994 or later, and (ii) all available dates of IEI diagnosis. We also assessed the impact of diagnostic timing and reverse causation, we performed analyses applying longer lag periods between IEI and HL diagnosis (≥2 and ≥5 years), in addition to the predefined ≥12-month lag before study entry. All analyses were performed using IBM SPSS Statistics (version 30.0 IBM Corp, 2024).

*References*

1.	Ludvigsson, J.F., et al., *External review and validation of the Swedish national inpatient register.* BMC Public Health, 2011. **11**: p. 450.

2.	Barlow, L., et al., *The completeness of the Swedish Cancer Register: a sample survey for year 1998.* Acta Oncol, 2009. **48**(1): p. 27–33.

3.	Brooke, H.L., et al., *The Swedish cause of death register.* Eur J Epidemiol, 2017. **32**(9): p. 765–773.

4.	Ludvigsson, J.F., et al., *The Swedish personal identity number: possibilities and pitfalls in healthcare and medical research.* Eur J Epidemiol, 2009. **24**(11): p. 659–67.

5.	Edwards, E.S.J, et al., *Beyond monogenetic rare variants: tackling the low rate of genetic diagnoses in predominantly antibody deficiency.* Cell Mol Immunol. 2021 **18**(3):588-603.

6.	Poli, M.C., et al., *Human inborn errors of immunity: 2024 update on the classification from the International Union of Immunological Societies Expert Committee.* J Hum Immun 2025; **1** (1): e20250003.

7.	Paganelli, R., *The early history of the ESID registry.* J Hum Immun 2026; **2** (2): e20250248.

8.	Kindle, G., et al., *Inborn errors of immunity: Manifestation, treatment, and outcome—an ESID registry 1994–2024 report on 30,628 patients.* J Hum Immun 2025; **1** (3): e20250007.
